# Moving pain management programmes into the digital age: development and evaluation of an online PMP for people with chronic pain

**DOI:** 10.3389/fpain.2024.1337734

**Published:** 2024-04-04

**Authors:** Katie Herron, Alison Bradshaw, Matthew Liptrot, Gina Wieringa, Kerry Mathews, John Wiles, Selina Johnson

**Affiliations:** ^1^ The Pain Management Programme, The Walton Centre NHS Foundation Trust, Liverpool, United Kingdom; ^2^ Manchester University NHS Foundation Trust, Manchester, United Kingdom; ^3^ Faculty of Health and Life Sciences, The University of Liverpool, Liverpool, United Kingdom

**Keywords:** online, remote, pain management programme, outcomes, quality of life

## Abstract

**Introduction:**

In response to Coronovirus Disease (COVID-19) health care restrictions, the pain management programme delivered group treatment digitally (OPMP). We aimed to: 1) evaluate pain related outcomes of the OPMP, 2) evaluate patient satisfaction and qualitive feedback of the OPMP and 3) compare OPMP outcomes with the pre-pandemic face to face (F2F) PMP outcomes.

**Methods:**

Age, gender, pain duration, occupational status, referral information and patient satisfaction data were collected. Pre- and post-treatment pain related outcomes were compared by calculating mean difference, benchmarking with effect size (Cohen's *d*) and determining clinically significant change (CSC) for OPMP and F2F PMP.

**Results:**

Two-hundred and thirty-seven patients provided outcome data, with 60 completing the OPMP and 177 completing the F2F PMP. OPMP patients were 10 years younger than the F2F PMP (44.8 vs 53.3), more were female (6.5:1 vs 2.8:1), more were working (45% vs 27%) and fewer were retired (3% vs 17%). The OPMP showed improvements comparable to the F2F PMP. Large effect size was reported across all outcome domains including objective physical outcomes. Eighty-one percent of OPMP patients were ‘extremely likely’ to recommend the programme but just over 50% of patients felt F2F would provide greater clinical benefits.

**Conclusion:**

The results support that OPMP is effective for carefully selected patients following a multidisciplinary team assessment however more complex cases still require F2F PMP.

## 1 Introduction

Face-to-face (F2F) pain management programmes (PMPs) have a long-established evidence base (1, 2) that shows improvements in disability, mood, and pain-related catastrophising (3) and are recommended by the UK Faculty of Pain Medicine (4).

In response to the Coronavirus disease 2019 (COVID-19) pandemic, our pain service paused F2F contact in response to the UK government guidelines and therefore had to deliver pain services online to maintain patient treatment (5).

Online pain management programmes (OPMP) guidance highlighted several considerations including managing risk in a patient's residence, confidentiality, supporting patient digital skills, providing electronic materials, and staff training for online working (6). This also flagged the need for robust evaluation of outcomes, patient satisfaction, cost effectiveness and patient involvement. Tetlow and Woodhead further identified vital components for successful online working in non-pain populations including maintaining group cohesion, awareness of therapist demand, preparation for technological challenges, patient safety, consent, and confidentiality (7). This guidance was used to develop our OPMP that utilised ‘Microsoft Teams’ software and complied with NHS confidentiality requirements. To ensure staff and patients felt confident using the Teams platform, training was provided.

Of the few studies available that have examined OPMP outcomes, there is preliminary evidence for improvements in perceived health status, level of disability, mood, confidence managing pain, level of pain, reduction in healthcare costs (8), and reduction in opioid use (9). A recent systematic review showed significant yet small effect sizes for pain intensity, health-related quality of life, and depression (10) across 11 studies of varied online delivery routes including modular and self-directed only.

We conducted a practice-based audit to understand whether the OPMP developed in our service during the pandemic was effective and to compare it with previous methods of treatment delivery.

## 2 Methods and materials

### 2.1 Study design and setting

This study utilised a practice-based audit design that compared baseline and post-treatment outcomes.

All PMP treatment was delivered by The Walton Centre NHS Foundation Trust; a specialist neurology and neurosurgical hospital that provides a pain service for adults with chronic pain across a catchment area of 3.5 million. Patients referred to the PMP, following triage, are invited to attend a multidisciplinary team (MDT) assessment clinic. This involves medical screening by a Pain Consultant and assessment by a specialist team comprising a Clinical Psychologist, Occupational Therapist, and Physiotherapist. Suitable patients following this assessment are offered a place on a PMP.

The outcomes were collected prospectively with patients providing consent for their outcomes and demographical data to be stored on the ethically approved “PMP Registry” to be used for research purposes (19/NW/0130). The F2F patients had the option to complete their outcome questionnaires by paper, while in clinic, or they could use the online Qualtrics XMTM system as used for all OPMP patients.

### 2.2 Participants

All patients referred to our specialist pain service are from anywhere in the UK and have a chronic pain diagnosis and are >17 years old. Inclusion criteria for PMP suitability comprised motivation from the patient to engage, and no other unstable medical conditions that would affect engagement. Exclusion criteria comprised rapidly deteriorating disease, first or third trimester of pregnancy, psychological or psychiatric difficulties independent of pain that would affect engagement, or inability to process or retain information. Those deemed suitable following the MDT assessment are offered a PMP.

Available outcome data were sampled from the OPMP group between September 2020 and August 2021 (*n* = 60) and from the 16-day PMP (16 PMP) F2F group between September 2018 and August 2019 (*n* = 177).

### 2.3 Intervention

All PMPs are group-based, multidisciplinary rehabilitative treatments informed by cognitive behavioural therapy (CBT) with additional third wave integrative approaches [acceptance and commitment therapy (ACT); mindfulness; compassion-focused therapy] to support people to live well with pain. The PMP is delivered by psychologists, occupational therapists, physiotherapists, with further input provided by pain consultants and nurses. Content comprises components as recommended in the clinical guidelines (11).

The F2F programme comprised four full days over six weeks amounting to 116+ hours of intervention. Group size was 12–16 patients. The OPMP delivered 60+ h of intervention over three half day sessions per week for 6 weeks with six patients per group. Prior to running the online programme, “Expert Patients” were consulted by our service. These were patients who had previously attended a PMP and had expressed an interest in future service development (*n* = 4). No financial compensation was provided. Feedback from patients highlighted that replicating the intensity of the F2F programme would be unrealistic. It was suggested that online sessions would be more tiring compared with F2F and could lead to lapses in concentration. It was additionally suggested that group sizes below four would cause difficulties in group dynamics. In response to this feedback and guidance, regular breaks were built into the programme and groups sizes of six were planned.

The OPMP content was based on the F2F PMP and reviewed to ensure it remained consistent with PMP guidelines (4), while considering the recommendations in the existing literature, patient feedback, and practical limitations such as lack of access to a gym. The OPMP was fully led by clinicians and interactive with handouts as well as the opportunity for one-to-one support prior to or alongside the programme. Home learning activities were put in place to further support patients’ engagement with content. In addition, an online system for the collation of outcomes was introduced.

The key themes covered in both PMPs, in line with guidelines, comprised pain education; value-based target setting; balancing activities, mindfulness, communication, work, and employment; supported exercise sessions, pilates, posture correction and stretching; managing thoughts and emotions; self-compassion and acceptance processes; managing flare ups; and maintaining change. To reduce the timetable to fit the OPMP schedule, some repeated session themes were omitted. A session on sleep was also omitted from the OPMP and patients were asked to refer to the handout.

### 2.4 Outcomes

Outcomes were collected at pre-treatment baseline (PMP assessment clinic) and post-treatment reassessment (the last week of the programme). Outcomes were also routinely collected at 6-month post completion of a programme which have not been reported in this study. All measures apart from those required for physical functioning, performance, and the Beck Depression Inventory (BDI) were transferred to the electronic survey system Qualtrics XM™ software (https://www.qualtrics.com).

#### 2.4.1 Pain experience

“Pain Intensity” was rated from 0 (no pain) to 10 (most intense pain imaginable) over the previous week. This commonly used, reliable, and valid method consistently demonstrates sensitivity to improvements associated with pain treatment (12, 13), “Pain Distress” was rated from 0 (no distress) to 10 (extremely distressed) over the previous week, which was measured as part of the pain experience (12).

#### 2.4.2 Coping and cognitive appraisal

The Pain Catastrophising Scale [PCS; (14)] is a 13-item self-report measure designed to assess catastrophic thoughts associated with pain. The respondents indicate the degree to which example thoughts are experienced when in pain using a 5-point scale ranging from 0 (not at all) to 4 (all the time) (15). Higher scores indicate a greater degree of pain catastrophising (0–52). A total score of >30 represents a clinically significant level of pain catastrophising. For this audit, the total score is considered. The PCS is reliable (16) with good test–retest reliability (14) used extensively in clinical practice and research.

#### 2.4.3 Self-efficacy

The Pain Self-Efficacy Questionnaire [PSEQ; (17)] is a 10-item questionnaire, developed to assess confidence in performing activities. The PSEQ covers a range of functions, including household chores, socialising, work, as well as coping with pain without medication. The PSEQ total score are reported within this study which ranges from 0 to 60. In general, scores above 40 indicate high levels of self-efficacy, while scores below 30 suggest low self-efficacy (18). Internal consistency is high with good test–retest reliability and validity (19).

#### 2.4.4 Emotional functioning

The Beck Depression Inventory II [BDI-II, (20)] consists of 21 items that assess intensity of depression, which correspond to the Diagnostic and Statistical Manual of Mental Disorders (DSM-IV) criteria (20). Each item is a list of four statements arranged in increasing severity relating to a particular symptom of depression scored from 0 to 3, which is summed to give a single score. We have reported total scores with a total score of 0–13 considered minimal range, 14–19, mild, 20–28, moderate, and 29–63, indicating severe depression (21). This measure has been used in a variety of medical populations including those with chronic pain showing good reliability and consistency (12, 21).

Due to licencing restrictions for using the BDI-II online, the Patient Health Questionnaire-9 [PHQ-9; (22)] was used as an alternative for OPMP patients. The PHQ-9 is the nine-item depression module from the full PHQ. The PHQ-9 scores range from 0 to 27 with each of the nine items scored from 0 (not at all) to 3 (nearly every day). The PHQ-9 total score that we have reported is divided into the following categories of increasing severity: 0–4 (none), 5–9 (mild), 10–14 (moderate), 15–19 (moderately severe), and 20 or greater (severe) (22).

#### 2.4.5 Physical functioning

The Sit to Stand (STS) test was used as a physical performance measure to evaluate physical function by counting the total number of times a person can stand up from a chair in 1 min unsupported (23). Research shows that this an appropriate method for measuring physical performance as part of physiotherapy assessments (24) with good test–retest reliability (25).

#### 2.4.6 Performance

The Canadian Occupational Performance Measure (COPM) assesses client-perceived changes in problems encountered in daily activities (26). This measure is spilt into two components: (1) performance and (2) satisfaction. The performance and satisfaction scores can be generated for up to five self-selected problem areas that are each scored out of 10 (0 indicating low ability with selected tasks and 10 indicating high ability). The average scores are calculated by summing individual problem scores and averaging them by the number of problems. We report the total score for both components. Validity is supported (26) and reliability established in an arthritis population (27).

Due to the COPM's electronic-use restrictions, we utilised the patient-specific functional scale (PSFS) for the OPMP (28). The PSFS is a self-reported, patient-specific measure, designed to assess functional change in five self-selected tasks that are each scored out of 10 (0 indicating low ability with selected tasks and 10 indicating high ability). The average scores are calculated by summing individual problem scores and averaging them by the number of problems. We report a total score. The PSFS has been shown to be valid and responsive to change in musculoskeletal conditions such as neck pain, cervical radiculopathy, knee pain, and low back pain (29, 30).

#### 2.4.7 Patient satisfaction and feedback (OPMP)

The end-of-treatment patient satisfaction survey, issued separately from the clinical outcomes measures, asked patients to state how likely they were to recommend the OPMP on a 5-point scale of “Extremely Likely,” “Likely,” “Neither Likely nor Unlikely,” “Extremely Unlikely,” or “Don't Know.” To understand the experience of attending the OPMP, patients were also asked to identify the pros and cons of the OPMP, which were grouped into themes for the purposes of presenting the data.

### 2.5 Analyses

All data were analysed using the Statistical Package for the Social Sciences (SPSS) software (v.16.0, SPSS Inc., Chicago, IL, USA). Descriptive statistics were calculated for demographical data comprising pain duration, age, sex, occupational status, and pain diagnoses in addition to all outcome measures per group. Owing to the unexpected finding of differences between the online and F2F groups, a two-tailed statistic test of difference (*t*-test of non-parametric equivalence) was calculated for pain duration and age. In addition, the *χ*^2^ goodness-of-fit test was applied to test for differences in categorical data including sex, diagnoses, and occupational status.

To determine the size of the pre and post differences in outcomes, we utilised two methods as recommended by Morley (31):

1) Benchmarking with Effect Size (ES) where the average effect size [Cohen's *d* = (M2 − M1)/SD]) of outcomes was compared with that expected in a randomised control trial of a PMP (32). Surpassing the benchmark suggests a “good enough” effect of treatment. Effect sizes were categorised as small (0.2–0.4), medium (0.50–0.7), and large (≥0.8). Established minimum effect size benchmarks were derived from Fenton and Morley's review of routine clinical treatments for chronic pain (using aggregated data across treatment characteristics) (32). “Pain Experience” was determined by Pain Intensity and Pain Distress (*d *= 0.37); “Coping and Cognitive Appraisal” were measured by PCS and PSEQ (*d *= 0.19); “Emotional functioning” was measured by BDI-II and PHQ-9 (*d *= 0.33); and “Physical Functioning” was measured by STS (*d *= 0.37). The PSFS and COPM do not have corresponding benchmarks.
2) Clinically significant change (CSC) determines if a patient's change score is “clinically significant” rather than “statistically significant.” CSC is determined if the change score meets the minimum change set by the baseline variance. There are varied approaches for calculating CSC including changes within 0.2 up to 2 standard deviations from the baseline group mean (33, 34). For the purposes of this audit, we will determine CSC if improvement (pre–post) is more than 1 standard deviation of the baseline group mean (online or face to face). This suggests that patients who fulfil the criteria for CSC have made substantial improvement as a result of treatment.

Outcomes of satisfaction and feedback data are reported as frequency and collated themes.

## 3 Results

### 3.1 F2F PMP

**Figure 1:**
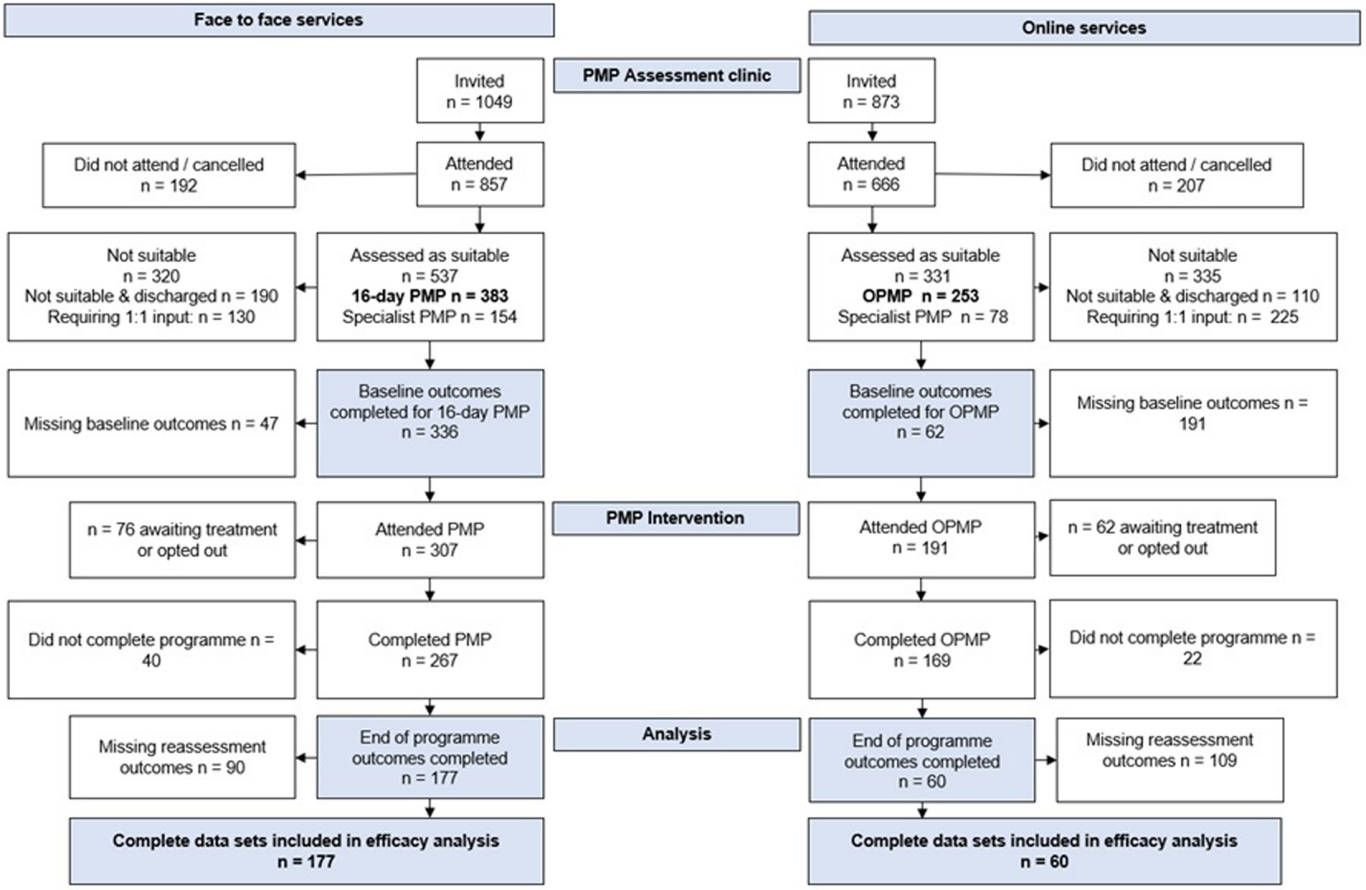
CONSORT diagram of study flow.

Between September 2018 and August 2019, 857 patients attended a PMP assessment clinic. Following the assessment, 537 (63%) were found suitable for a PMP intervention, with 383 (45%) being suitable for the 16-day F2F PMP. In total, 307 patients attended the programme and 267 (87%) completed the programme, with 177 (58%) complete data sets comprising baseline and reassessment available for efficacy analysis (Figure 1). For F2F PMP, the 12.3% drop-out rate during the programme was due to life circumstances, alternative treatment options pursued, or the patients choosing not to continue.

### 3.2 Online PMP

During the pandemic, September 2020–August 2021, 666 patients attended a PMP assessment clinic. Following the assessment, 331 (50%) were found suitable for a PMP intervention, with 253 (38%) being suitable for the OPMP. Totally 191 patients attended the programme and 169 (88%) completed the programme, with 60 (31%) complete data sets available for efficacy analysis (Figure 1). For these patients, the drop-out rate was 11.6% for the same reasons as stated previously.

Those suitable for specialist PMP were not included in the analyses. Missing outcomes were due to patient refusal to complete, patient did not attend the reassessment session post the treatment, or temporary lack of staff for follow-up completion. A large proportion of patients who did not complete the clinical outcomes required for efficacy analysis still returned the satisfaction survey.

### 3.3 Demographic characteristics

Demographical data are presented in Table 1. The average age for persons attending the OPMP was 44.8 years, which was statistically younger than those on the F2F programme at 53.3 years (*t* = −4.4; df = 84.4; *p* < 0.001). The number of male patients attending an OPMP was 8 (15%); when compared with the 47 (36%) on the 16-day PMP, this was statistically different (*χ*^2^= 4.4, df = 1; *p* = 0.04). Pain duration was not significant between the groups (*U* = 4,481.5; *p* = 0.66).[Table T1]

**Table 1 T1:** Demographical data per group.

	OPMP (*n* = 60)	F2F PMP (*n* = 177)
Age, mean (min; max)	44.8 (18; 76)	53.3 (25; 75)^a^
Sex (female:male)	52:8	130:47^a^
Pain duration (months)	106.6	124.2
Occupational status (%)		
Full-time work	27	15
Part-time work	18	12
Not working due to pain	32	34
Not working: other reasons	2	5
Not working: other health condition	0	12
Homemaker	0	4.3
Student	7	0
Retired	3	17
Voluntary work	0	1
Diagnoses (%)		
Fibromyalgia	25	21
Chronic widespread pain	18	19
Chronic low back pain	25	31
Neck pain	7	6
Other pain diagnoses (≤5%)	25	23

OPMP, online pain management programme; 16 PMP, 16-day face-to-face pain management programme; *n*, number of patients.

^a^ Significant difference between groups (*p* ≤ 0.05).

The percentage of patients who were working full or part time attending an OPMP was significantly more at 27% when compared with the F2F PMP at 15% (*χ*^2^ = 3.8, df = 1; *p* = 0.05). The F2F 16-day PMP had 17% retirees whereas as the OPMP had only 3%; however, this did not reach statistical significance (*χ*^2^ = 0.9, df = 1; *p* = 0.32). The pain diagnoses of the online and F2F groups were similar, with about 75% experiencing spinal pain or widespread pain. The most common diagnoses for both groups were Fibromyalgia, Chronic Widespread Pain, and Chronic Low Back Pain.

### 3.4 Baseline mean outcomes

All PMP outcome data are presented in Table 2. At baseline, moderate pain intensity and pain distress scores were reported for both the programmes. Both programmes were close to a clinically significant level of pain catastrophising >30. The PSEQ scores for both programmes were >30, suggestive of low self-efficacy. At baseline, the F2F PMP patients fell within the criteria for “moderate” depression on the BDI (26.9), and those in the OPMP were in the “moderately severe” range according to the PHQ-9 (14.1). The F2F PMP patients were able to complete 5.7 unsupported sit–stands compared with the score of 4.9 for patients on the OPMP. For pain-free persons dependent on age, a score of 20+ would be expected (24). The F2F PMP patients reported 2.9 for COPM performance and 2.1 for satisfaction. On the OPMP, the average baseline score for PSFS was 2.8. This indicates a low level of performance for both populations in relation to self-selected tasks.

**Table 2:** Change in outcome variables pre and post intervention.

		Pre		Post		Pre–Post change			
		Mean	SD	Mean	SD	Mean diff.	% impro.	ES	CSC
Pain Int.	OPMP	7.3	1.8	5.7	2	1.6	22%	0.9	48%
	F2F PMP	7.4	2	5.1	2.3	2.3	28%	1.1	58%
Pain Di.	OPMP	7.1	1.6	5.3	2.1	1.8	26%	1	53%
	F2F PMP	7.5	1.4	6.5	1.8	1	13%	0.7	32%
PCS	OPMP	29.1	9.9	21.2	11.2	7.9	27%	0.8	43%
	F2F PMP	30.4	11.1	19	12.1	11.4	36%	1	48%
PSEQ	OPMP	16.3	10.2	28.2	12.6	−11.9	73%	1.2	50%
	F2F PMP	17.8	9.5	31.4	11.3	−13.8	77%	1.5	59%
BDI-II	OPMP	—	—	—	—	—	—	—	—
	F2F PMP	26.9	10.8	17.5	11.5	9.3	35%	0.9	43%
PHQ-9	OPMP	14.1	5.1	10	5	4.1	29%	0.8	35%
	F2F PMP	—	—	—	—	—	—	—	—
STS	OPMP	4.9	5.1	9.5	7.5	−4.6	95%	0.9	42%
	F2F PMP	5.7	5.5	9.7	5.4	−4	71%	0.7	32%
COPM-P	OPMP	—	—	—	—	—	—	—	—
	F2F PMP	2.9	1.7	5.8	1.6	2.9	100%	2.46	76%
COPM-S	OPMP	—	—	—	—	—	—	—	—
	F2F PMP	2.1	1.2	5.6	2	3.5	167%	3.12	86%
PSFS	OPMP	2.8	1.3	5.8	1.5	3	107%	2.3	91%
	F2F PMP	—	—	—	—	—	—	—	—

Mean Diff., mean difference; % impro., percent improvement; ES, effect size; CSC, clinically significant change; Pain int, pain intensity; Pain di, pain distress; PCS, pain catastrophising scale; PSEQ, pain self-efficacy questionnaire; BDI-II, Beck depression inventory II; PHQ-9, patient health questionnaire; STS, sit to stand; COPM-P, Canadian occupational performance measure-performance; COPM-S, Canadian occupational performance measure-satisfaction; PSFS, patient-specific functional scale.

Due to the unexpected differences observed between the F2F PMP and OPMP demographics, we performed a test of difference between baseline scores for these groups. No significant differences were observed at baseline.

### 3.5 Benchmarking with effect size

The outcomes for the OPMP group all surpassed the expected benchmarks for all the PMP outcome domains outlined in Fenton and Morley (32). Effect sizes reported for the OPMP group were for the pain experience (Pain Intensity 0.9, Pain Distress 1.0), coping and cognitive appraisal (PCS 0.8, PSEQ 1.2), emotional functioning (PHQ-9 0.8), and physical functioning (STS 0.9) (Table 3). The F2F PMP group also surpassed all benchmarks. Effect sizes reported for the OPMP for each domain that correspond with those outlined by Fenton and Morley were as follows; pain experience (pain intensity 0.9, pain distress 1.0), coping and cognitive appraisal (PCS 0.8, PSEQ 1.2), emotional functioning (PHQ-9 0.8), and physical function (STS 0.9) (Table 3).

**Table 3:** Patient ratings for likelihood of recommending OPMP to others.

	*N* (167)	%
Extremely likely	137	81
Likely	26	15
Neither likely nor unlikely	3	2
Extremely unlikely	1	0
Don’t know	2	0

*n*, number of patients; %, percentage of patients.

### 3.6 Percentage improvements

Comparing groups directly for similar measures (Table 2), the percentage of improvement did not differ more than 4%–9% between the OPMP and the F2F PMP groups across all measures apart from pain distress and STS, where the OPMP group showed 13% and 24% greater improvement, respectively.

### 3.7 Clinically significant change

For all outcome domains, more than 32% of patients reported CSC on both programmes. In relation to pain experience, 48% of patients on the OPMP reported CSC in respect to pain intensity compared with the 58% for the F2F PMP, and 53% of patients on the OPMP reported CSC in respect to pain distress compared with the 32% for the F2F PMP. For coping and cognitive appraisal, 43% of patients on the OPMP reported CSC in respect to PCS compared with the 48% for the F2F PMP and 50% of patients on the OPMP reported CSC in respect to PSEQ compared with the 59% for the F2F PMP. Within the emotional functioning domain, 43% of patients on the OPMP reported CSC in respect to BDI, while 35% of patients reported CSC for PHQ-9. For physical functioning, 42% of patients demonstrated CSC for STS compared with 32% for the F2F PMP. For the COPM, 76% and 86% of patients recorded a CSC for performance and satisfaction respectively within the F2F PMP. A similar measure used to assess task performance within the OPMP showed 91% of patients reported a CSC.

### 3.8 Patient satisfaction

Table 3 shows that (81%) who attended the OPMP would be “extremely likely” to recommend it to others.

### 3.9 Patient feedback

Table 4 presents the themes of pros and cons of attending an OPMP as written in free text by patients who completed the satisfaction and feedback questionnaire. While there were clear positive benefits to attending an OPMP, 33 patients reported a preference for F2F sessions whereas only 12 preferred online. The patient feedback suggests that the online programme did allow them to overcome some practical barriers to attendance; however, they feel greater clinical gains, especially from a physiotherapy and social interaction perspective, could be made with F2F programmes.

**Table 4:** Themes of pros and cons for attending an OPMP ranked in order of number of patients who all reported the same theme; note, patients could provide more than one answer and therefore numbers are not cumulative.

Pros	*n*	Cons	*n*
Avoiding travel was a benefit	57	Reduced social aspect affecting group discussions and support	31
Relaxed/less stress being at home	18	Physio sessions tricky/less effective/cannot use equipment	28
Easier to manage work/childcare	11	No hydro	8
Practically easier to attend	7	Would prefer to meet the people face to face	7
Smaller groups more manageable	6	Poor internet/tech probs (staff and pts)	6
Easier to access but perhaps F2F is better	4	Less informal conversation with the group	3
More able to open up being in home environment	4	Communication is difficult online/talking over each other	2
Less physically challenging so managed to attend	3	No physical reassessment	2
Was sceptical but found it really helpful	2	Difficult to concentrate on screen for that long	2
Future course could mix online and face-to-face interactions	2	Felt a bit rushed	1
Less fatiguing/worried 16 full days would be too much	2	Less emotionally connection	1
Didn’t miss any days as the half days were manageable	1	No structured group time without clinician being there	1
Its reasonable given the current circumstances	1	Less able to talk about distress online	1
Online is probably cheaper	1	Listening online is harder with hearing impairment	1
It’s good if you’re comfortable with technology	1	Felt disconnected and not able to concentrate for meditation	1
Got seen in good time	1	Felt less support from the clinicians	1
Reduced long waits to see health professionals	1	Physio difficulties only picked up in final week	1
Everything accessible	1	Felt diluted	1
		Easier to get distracted at home	1

## 4 Discussion

The development of an OPMP enabled our department to deliver services throughout the pandemic. This audit shows that the OPMP surpassed the established benchmark expected in F2F PMPs for pain experience, cognitive appraisal, emotional impact, and physical functioning (32). Furthermore, the outcomes were largely comparable with our previous F2F programme outcomes (16-day PMP). It is important to highlight that the OPMP groups were younger, more were women, and more likely to be in work compared with the F2F PMP group. This suggests OPMPs are attended by a different population compared with the F2F groups. Unlike the systematic review that showed only significant improvements in outcomes across the 11 studies using online programmes that did not meet the standard for clinically significant change (10), this study presents more promising findings. This may also reflect the benefits of a directly clinician-led online programme as opposed to a modular or self-directed one.

The differences between OPMP and the F2F PMP for age and working status could be due to the selection of those motivated and able to have their treatment online. In addition, the MDT assessment determined likely benefits from the OPMP. It is our clinical experience that those deemed unsuitable for online PMP were more likely to have complex psychosocial and poorer functioning presentations that required F2F treatment. This suggests that our OPMP is being accessed by a highly selected group that is different to our usual F2F group.

Compared with the pre-pandemic F2F groups, we observed lower opt-in rates for OPMP assessments. We speculated that this was because patients had concerns about online appointments and if they offer the same benefits as F2F. This has also been reported elsewhere where patients expressed concerns on how OPMPs could foster sharing of experiences and sense of support (6). In addition, patients were concerned that online sessions reduce opportunities for individualised intervention and access to gym facilities and exercise equipment (6). While our OPMP factored in these concerns through the use of breakout rooms, one-to-one session with team members, and adapted exercise plans, at the point of opting in for an online assessment, these adaptations would not have been discernible to patients. This suggests that staff should explicitly address patient concerns on the OPMP.

The outcomes suggest that OPMPs did not disadvantage outcomes compared with F2F service delivery. The viability of online interventions in supporting individuals to cope with and self-manage has been indicated across different chronic illnesses (35), and a recent systematic review of web-based interventions for chronic pain have highlighted the potential of online CBT-based programmes in reducing catastrophising and improving patients’ attitudes (36). Similar results have been observed for approaches based on ACT principles; Herbert et al. (37) observed that an eight-week internet-delivered acceptance and commitment therapy was as effective as an in-person delivered intervention in improving pain-related disability immediately after and at six months post intervention.

Specifically, outcomes for F2F or online groups were largely comparable apart from for pain distress and sit-to-stand measures, where the OPMP patients achieved at least a 10% higher average for CSC. We speculate that the F2F group comprised people of varied complex needs who would not have been suitable for the OPMP although the baselines were similar between the groups. It is therefore likely that those higher functioning patients in the OPMP had greater scope to improve physically and be related to a younger age. It could also be due to the change in how the sit-to-stand test is conducted. There is potential that patients find it easier to do a sit-to-stand test without having travelled to the hospital and therefore may have greater capacity to achieve a higher score at home.

The F2F group showed greater clinically significant improvements for pain intensity. It is possible that the imposed lockdown conditions that patients completing our OPMP were living under may have influenced the potential for change in pain intensity. The physical and social distancing measures warranted by the pandemic may have impacted pain symptoms through changes in physical activity levels, worsened mood, and greater levels of anxiety (38)—changes that would not have been reflected in initial outcome measures given these were taken during the UK lockdown. Fallon et al. (38) observed that individuals with higher levels of pain catastrophising perceived greater increases in pain intensity during the lockdown period. OPMP patients with high levels of pain catastrophising may have perceived greater increases in pain intensity during the lockdown period when compared with the subgroup of patients who completed the F2F programme prior to the pandemic, thus mitigating improvements in pain intensity as a result of our OPMP.

Although the OPMP and the F2F PMP scores for depression cannot be compared directly due to differing measures, both the BDI-II and PHQ-9 have a “moderate” range within their scoring categories. Given that a large number of patients fell into the moderately severe range from the OPMP, this could tentatively suggest that patients in the online group were more depressed. We would suggest exploring the effectiveness of PMP interventions in future studies considering direct comparison of such measures to compare any differences as a consequence of the outcome measures used. The higher prevalence of moderately severe depression in the online group may reflect the emotional impact of the pandemic on people with pain, as demonstrated elsewhere (38).

It is also important to note that the majority of patients who took part in the feedback survey report a preference for F2F over online PMP. While the OPMP presented practical benefits, such as reducing burden on travel or demands from other commitments, the patients felt more clinical gains could be made with F2F PMPs, such as physiotherapy access and interaction with other patients. Therefore, while online platforms present an opportunity to deliver treatment with potentially less resource requirements, the potential value of physical interaction voiced by strong patient feedback should also be recognised.

A major limitation of the current audit is the large number of missing baseline outcomes that were available for our OPMP. The low number of collated baseline outcomes was attributable to delays and organisation of data collecting following the onset of remote working and staffing changes. This has been an important learning objective for the study team, and the importance of outcome data collection protocols, staff training, and data monitoring have since been put in place. A drawback for this audit was that the analysed outcomes were disproportional between the two interventions. While this is less than ideal, the chosen methods of analysis were effect size and CSC, which are less likely affected by different group sizes compared with other potential measures of effect such as between group t-tests, where the imbalance in group size would have been more confounding. Where tests of difference were conducted, variability in the data enabled determination of test statistics.

While there are concerns about the limited theoretic foundation of OPMP development (39) and limited stakeholder involvement (40), our OPMP involved consultation with PMP guidelines, clinical experience, and consultation with relevant stakeholders to prioritise where adaptations were required (4, 7) to ensure that our virtual delivery is aligned with evidence-based treatment. These components include “skills training and activity management”; “cognitive therapy methods”; “education”; “graded activation guided by participant goals”; “methods to enhance acceptance, mindfulness, and psychological flexibility”; “physical exercise”; and “graded exposure”. With the lack of published guidance on providing group-based PMP using virtual means, while enabling interaction between that group participants and healthcare providers, it is hoped that this audit shows a likely feasible model of delivery with encouraging outcomes.

The differences in outcome collection for the F2F PMP and OPMP limit the degree to which we can compare outcomes. F2F patients completed the questionnaires while in the department with the support available and this ensured all items were completed. OPMP patients completed the online questionnaires without direct support apart from the sit-to-stand test, COPM, and PSFS. This has also resulted in a reduction of completed baseline assessments due to missing questionnaires or items. In addition, licencing restrictions of pre-pandemic questionnaires, such as the BDI-II, meant we had to use alternatives.

The audit also lacked the 6-month follow-up data for online patients and therefore we do not know the long-term efficacy of any gains made as a result of the treatment. Obtaining follow-up results from virtually delivered programmes should also be a primary focus of future research.

The authors have demonstrated that in terms of limited outcome, an online version of pain management may be viable and worth pursuing, but short-term benefits are almost always demonstrable in those who have been selected as suitable and who have not dropped out, and independent longer-term evaluation is needed to counter social desirability bias.

The attempt to identify the pros and cons of attending an OPMP using free-text feedback is helpful, but independent qualitative interviews would have been persuasive.

To the best of our knowledge, this audit is one of the first to directly compare findings on a range of outcome measures deemed as important in the evaluation of PMP delivery with a direct comparison with pre-pandemic F2F PMPs. Research examining the use of online self-management interventions is in its infancy and a number of limitations have already been identified, one of which is limited evidence of efficacy (41). In light of radical changes to service delivery, and recent findings from the NHS Innovation Agency report (42) that support the continued use of a hybrid model, obtaining comparable outcome measures to aid the evaluation of online programme efficacy is essential.

Online PMPs have the potential to reduce health inequalities related to poor availability of services, mobility concerns, or limited financial resources to support travel and parking issues (43). Conversely, they may add to health inequalities if people lack the resources to access treatment or have complex social situations that prevent them from engaging with therapy within their home environment. Further barriers have been described as a more distanced relationship with clinicians for those who struggle to work through the material (44) and potential safety concerns for some individuals regarding engaging in physical activity at home. Further research is needed to understand the needs of patients and their perceived barriers to accessing and supporting treatment. Further research is also required to better understand the needs of individuals receiving online interventions, those who opt out of treatment, patient selection, and the most optimal way of providing digital care (45).

## 5 Conclusion

This audit provides the first comparative evidence of OPMP and F2F PMP delivery. Outcomes support the use of OPMP that includes content as recommended by core PMP standard guidelines. Patients found suitable for an OPMP differ from those found suitable for F2F PMP. Therefore, online content cannot be considered an exclusive alternative to traditional F2F delivery. We recommend the inclusion of both online and F2F PMPs following careful and considered patient selection and further research to inform and standardise content.

## Data Availability

The raw data supporting the conclusions of this article will be made available by the authors, without undue reservation.
